# P65. Minor-histocompatibility-antigen UTY as target for graft-versus-leukaemia and graft-versus-haematopoiesis in the canine-model

**DOI:** 10.1186/2051-1426-2-S2-P39

**Published:** 2014-03-12

**Authors:** D Bund, FG Gökmen, J Zorn, R Buhmann, HJ Kolb, H Schmetzer

**Affiliations:** 1 University of Munich-Grosshadern, Haematopoietic Cell Transplantation MED III, Munich, Germany; 2 Helmholtz Center Munich, CCG Haematopoietic Cell Transplantation, Munich, Germany

## Background

In haploidentical-SCT male-patients with female-donors have better prognosis compared to female-to-male-combinations due to Y-encoded minor-histocompatibility-antigens recognised by female-allo-immune effector-lymphocytes in the context of a graft-versus-leukaemia-(GvL)-effect. We provide data in a dog-model that the minor-histocompatibility-antigen UTY might be a promising target to further improve GvL-immune-reactions after allogeneic-SCT.

## Materials and methods

Canine (c) purebred-beagle-dogs’ PB and BM were studied. T2-cells (HLA-A2+, TAP-deficient) were used. These human-(h)-UTY-sequence-derived HLA-A2-binding-peptides were investigated: W248 (WMHHNMDLV), T368 (TLAARIKFL), K1234 (KLFEMIKYC). ***In vitro:*** Autologous-cDCs were generated with best of three DC-methods (*Calcium-Ionophore*, *Picibanil*, *Cytokines*). Generation cUTY-specific-CTLs: CD3+ T-cells were co-cultured with autologous-mature cDCs+hUTY-peptides (weekly restimulation for 21 days; +hIL-2, +hIL-7). Cytotoxicity and antigen-specificity were determined by [^51^Cr]-release- and cIFN-g-ELISPOT-assays. Cells were quantified day 0 and of harvest using anti-cmAbs/hmAbs (FACS), UTY-mRNA-expression via RT-PCR-analysis. ***In vivo*:** A female-dog was immunised with PBMCs from a DLA-identical-male-dog (day 0 and 14). PB-derived T-cells were harvested 35 days post 2^nd^-injection followed by analysing UTY-specific-reactivity.

## Results

Female cUTY-specific-CTLs were stimulated *in vitro* using autologous-DCs loaded with three HLA-A2-restricted UTY-derived-peptides (≤2.9-fold-expansion) and specific T-cell-responses were determined in 3/6 female-dogs. CTLs specifically recognised/lysed autologous-female peptide-loaded-DCs (900 spots/100,000 T-cells (median)/≤47.9%), but not naive autologous-female-DCs and -monocytes (p≤0.026). They mainly recognized BM and to a lower extent DCs, monocytes, PBMCs and B-cells from DLA-identical-male-littermates and peptide-loaded T2-cells in an MHC-I-restricted manner (up to p≤ 0.046). UTY-mRNA was only expressed in male-cells. A UTY-/male-specific-reactivity was also obtained *in vivo* after stimulation of a female-dog with DLA-identical-male-PBMCs.

## Conclusions

We demonstrated natural UTY-processing/presentation in dogs. Female-dog-CTLs were specifically stimulated by HLA-A2-restricted-UTY-peptides, thereby enabling recognition of DLA-identical-male-cells, mainly BM-cells. These observations suggest UTY as a promising candidate-antigen to improve GvL-reactions in the course of immunotherapy. Next-generation-sequencing and specialised-bioinformatics-algorithms are now focus for human-individualised-leukaemia-treatment (T-cell-receptor-Profiling, detection/selection of T-cell-receptor-clones or DC-based-immunotherapies).

